# Preventive Medicine

**Published:** 1879-10

**Authors:** 


PREVENTIVE MEDICINE.—We have had frequent occasion to remark, that one of the greatest obstacles in the way of real progress in any of the departments of medical science, is the disposition of writers and investigators to accept mere opinions as facts, and to draw inferences or conclusions from an inadequate number of observations, or from observations very imperfectly made.

In no department is this more strikingly illustrated at the present time, than in that of etiology in its relations to sanitary science or preventive medicine. The close relation of this department to municipal authorities, and measures for the promotion of the public health, makes it specially desirable that the profession should avoid indulgence in mere theories and hasty inferences, and should exercise the utmost patience and caution in the investigation of all practical questions, and scrupulous accuracy in the relation of facts, and the expression of such conclusions as affect the public interests. More especially should this rule be observed by such members of the profession as hold official positions as health officers, and members of the Health Boards, whether municipal, State, or national; because to them the public look for guidance, and justly expect that the opinions they express, and the measures they propose, will fairly and accurately represent the present status of medical knowledge. A failure in these particulars, has led, in some instances, to the most extravagant assertions as to what sanitary measures can accomplish in the prevention of disease; in others, to stories about disease producing *germs* in everything we eat, drink, or wear, more alarming or sensational to the public ear, than the story of the Arabian Nights to the ear of credulous childhood; and in still others, to the most reckless inaccuracies in the statement of facts, and the most tenacious adherence to preconceived theories though confronted by a thousand facts wholly irreconcilable with them. One of the most common extravagances here alluded to, is the often repeated claim of sanitarians that they can, not only, moderate the severity and lessen the prevalence of a large class of diseases including scartlatina, diphtheria, etc., but can by sanitary measures "stamp them out," or eradicate them from the community. Yet who has ever seen the promise fulfilled? In regard to the sensational stories about disease-producing germs, if the people really believed a fractional part of what has been written and published in medical journals and newspapers and duly illustrated with cuts, they would properly fear to breathe or eat, or even travel on the same highway over which a funeral cortege had once borne a body dead from scarlet fever. But the practice that does most to retard real progress is the constant exhibition of carelessness and inaccuracy in the statement of facts, not only in newspaper reports and ex-parte gossip called "interviews," but in medical journals and official documents. Take as a specimen, the following paragraph from the first page of the *National Board of Health Bulletin* for September 13th, 1879:

"Last year the intensely infected boat, John B. Porter, was an unchallenged rover on the Mississippi and Ohio rivers, and scattered pestilence broadcast for a distance of 500 miles." Such a statement in such a paper, is liable to be quoted by writers through all coming time, as reliable proof that the said boat did actually rove unrestrained and recklessly along the Mississippi and Ohio rivers, and did actually scatter the pestilence, yellow fever, broadcast, that is in every direction, for a distance of 500 miles.

But what were the facts? If there was any truth in the statement made at the time concerning the progress of that boat, instead of roving unchallenged about the Ohio, it was absolutely prohibited from landing on either shore, from the mouth of the Ohio river to Galliopolis (save at Louisville), and its unfortunate living freight was thus compelled by municipal and sanitary authorities, to keep the middle of the river until there were not enough left alive and well to direct the craft further, and they tied it up at Galliopolis, from inability to do otherwise. And where was the *pestilence* that it "scattered broadcast for the distance of 500 miles?" Can the writer of that sentence point to a single case that contracted the fever from that boat on either side of the Ohio from Cairo to Galliopolis except at the latter place where it was obliged to stop? Did any resident of Louisville take the disease from its landing there? We are told by those who ought to know that there was not one. We have not made this quotation from the *Bulletin* for the purpose of criticising any of the measures proposed by the National Board of Health; but solely as a specimen of the reckless inaccuracy of statements with which our current literature concerning epidemics, is filled; and which constitute one of the most serious obstacles in the way of all reliable investigations. Simply because they render it impossible for the investigator to know, in many instances, whether he is collating actual facts or mere fictions.

Another evil of hardly less magnitude, consists in the adoption of a particular theory concerning the etiology of a disease, and the mode of its propagation, and then doggedly rejecting every fact that appears to militate against the theory. The following will more perfectly illustrate our meaning. In the Cincinnati *Lancet and Clinic* for the 6th inst., a case of suspected yellow fever is reported, and a distinguished health officer's opinion requested concerning the diagnosis. In his reply we find the following paragraph: "The high temperature towards the last (103° F.), the yellowness of the skin, suppression of urine, and presence of albumen, are certainly strong points in favor of the supposition that the case was one of yellow fever, *nevertheless as there is no possible means of determining whether the patient was exposed to infection, the case must be overshadowed by some doubt, as such a thing as sporadic yellow fever*, or *fever developing de novo is out of the question.*" In other words, the *diagnosis*, instead of being based on the symptoms and pathological changes, is made to depend on the ability or inability to trace exposure to infection, and one of the most important questions now awaiting solution by the unbiased investigator, namely, whether yellow fever can originate sporadically or not, is dogmatically ignored. Of course, men guided by such a spirit of inquiry, will collate and credit only such facts as appear to prove their theory. Those who are predetermined to prove that the yellow fever in our country is always received from contact with infection or contagion, will take, for instance, the facts connected with the behavior of the disease at the quarantine in St. Louis in 1878, where the physician engaged in inspecting the boats coming up from New Orleans, Memphis, etc., took the disease and died, together with several of the hands employed on the ferry-boat carrying patients from infected boats to the quarantine hospital; and wherever a case occurs, the nature of which cannot be denied, and no connection with infection is apparent, they will fill the gap by *imagining* that some fruit, or rags, or something had been brought into the neighborhood, or that the contagious germs had been lying latent from some previous year. While those predetermined to maintain the opposite extreme of absolute non-contagion, will seize with equal avidity the facts connected with the behavior of the disease the same season at Louisville, where refugees from Memphis and all other infected places, were admitted with the utmost freedom, and where many got sick with the fever after their arrival, some in hotels, some in private houses, and many were taken to a special hospital under the care of Dr. Marvin, where they were attended by over twenty nurses, and were freely visited by physicians, clergymen, and even ladies, and not one of all these contracted the disease. Thus the advocates of each theory have abundant proof to sustain them, and to maintain the same controversy that was waged with so much vehemence in the days of Rush and Hosack. How much better it would be for the credit of the profession, and how much more rapid would be our progress in arriving at reliable practical results, if each one would hold his theories and preconceived opinions so far in abeyance, as to enable him to receive and give due weight to every well ascertained fact concerning the origin and propagation of any disease.

And where facts appear to some extent contradictory or pointing towards opposite conclusions, like those to which we have just alluded, concerning the yellow fever in St. Louis and Louisville in 1878, instead of using them to maintain opposite sides of a controversy, let us be more logical and assume that if persons in free communication with the yellow fever in quarantine near St. Louis contracted the disease, while a larger number and variety of persons in free communication with the same disease in the quarantine hospital at Louisville, all escaped, there must be some difference either in the habits and susceptibilities of the persons exposed in the two places, or else in the local and meteorological conditions of the places themselves. Therefore, just what constituted such differcnce becomes at once the paramount subject for further investigation.

That yellow fever, cholera, and all similarly epidemic and apparently infectious diseases, propagate themselves with great readiness in some localities and not in others, at some seasons of the year and not at others, is a matter of history too plain to be denied. If this is granted, then the next step is, for all officers of health, sanitarians, and physicians, to enter patiently, minutely and perseveringly, into an investigation of the reasons for such differences. The geological, topographical, meterological, and sanitary conditions of each locality, together with the habits and social condition of the people, must be studied from year to year, including alike seasons of sickness and health. By this method, and this only, can we ever hope to determine positively the efficient cause of any given epidemic disease, and the conditions on which its propogation depends. We think the members of the National Board of Health have given good evidence of a fair appreciation of the nature of the work before them; but many of the State and local boards need to be more cautious about enforcing upon the people burdensome and arbitrary measures founded on mere theoretical assumptions, and to exercise more patience and diligence in availing themselves of the results of past experience, as well as in devising and executing well considered methods of further investigation. And, above all, do we want more care and accuracy in the observation and record of facts.

